# Effects of Diet and Altitude on the Microbiota of the First Compartment of the Stomach in Peruvian Alpacas in Highland Puna Regions and Their Correlations with Blood Parameters

**DOI:** 10.3390/microorganisms14010138

**Published:** 2026-01-08

**Authors:** Nils H. Flores-Huarco, Richard Estrada, Yolanda Romero, Pedro Coila, Diana Sanchez, Jorge L. Maicelo, Wigoberto C. Alvarado, Carlos Quilcate, Mery Aliaga, Walter Galindo, Jorge Saavedra-Ramírez, Henry Apaza, Carlos I. Arbizu

**Affiliations:** 1 Unidad de Posgrado en Ciencia Animal, Universidad Nacional del Altiplano de Puno, P.O. Box 291, Puno 21001, Peru; dbionils@gmail.com; 2 Instituto de Investigación en Bioinformática y Bioestadistica, La Molina, Lima 15024, Peru; yolanda.bioinfo@gmail.com; 3 Dirección de Desarrollo Tecnológico Agrario, Instituto Nacional de Innovación Agraria (INIA), Av. La 11 Molina 1981, Lima 15024, Peru; ceqp2374@yahoo.com; 4 Facultad de Medicina Veterinaria y Zootecnia, Universidad Nacional del Altiplano de Puno, P.O. Box 291, Puno 21001, Peru; pcoila@unap.edu.pe (P.C.); mlaliaga@unap.edu.pe (M.A.); waltergalindos@gmail.com (W.G.); hapazaa@epg.unap.edu.pe (H.A.); 5 Escuela Profesional de Medicina Veterinaria, Universidad Nacional de San Antonio Abad del Cusco, Cusco 08003, Peru; diana.sanchez@unsaac.edu.pe; 6 Facultad de Ingeniería y Ciencias Agrarias, Universidad Nacional Toribio Rodríguez de Mendoza de Amazonas (UNTRM), Cl. Higos Urco 342, Chachapoyas 01001, Peru; jmaicelo@untrm.edu.pe (J.L.M.); wigoberto.alvarado@untrm.edu.pe (W.C.A.); 7 Facultad de Ingeniería, Universidad Nacional Autónoma de Alto Amazonas, UNAAA, Calle Prolongación Libertad Nro. 1220-128, Loreto, Yurimaguas 16501, Peru; jsaavedrar@unaaa.edu.pe

**Keywords:** alpaca, first compartment stomach, Puna environments, 16S/18S, changes

## Abstract

This study explores the intestinal microbiota of eight 18-month-old male alpacas from two distinct high-altitude regions in Peru: the Wet Puna (4200 m above sea level) and the Dry Puna (4900 m above sea level). Using 16S rRNA and 18S rRNA metabarcoding, microbial communities of bacteria, archaea, fungi, and protists were analyzed from the first compartment of the stomach (C1) to investigate the diversity, taxonomic composition, and correlations with hematological parameters. Significant differences in microbial diversity and composition were observed between regions, driven by dietary and environmental factors. The Wet Puna exhibited greater alpha diversity in bacterial and fungal communities, while beta diversity highlighted distinct microbial compositions. Key taxa, such as *Prevotella ruminicola* and *Acetitomaculum*, were associated with energy metabolism and host adaptation, whereas methanogenic archaea (*Methanobrevibacter*, *Methanosphaera*) dominated in the Dry Puna, reflecting adaptations to arid conditions. Correlations between microbial taxa and hematological variables, such as *Acetitomaculum* with red blood cell count and *Eremoplastron* with neutrophil percentage, emphasize the complex interplay between microbiota and host physiology. These findings contribute to understanding microbial adaptations in high-altitude livestock and provide practical insights for enhancing alpaca management and conservation strategies through tailored nutritional approaches and sustainable grazing practices.

## 1. Introduction

Alpacas (*Vicugna pacos*) are native to the Andean region of South America and hold significant cultural and economic importance due to their historical and ancestral heritage in Peru. These animals, particularly the Huacaya breed, are highly valued for their high-quality fiber, which is in demand in the textile industry [1,2,3]. Their exceptional adaptation to the Andean highlands, specifically the Puna region, highlights their importance as a genetic resource in environments characterized by scarce and low-nutritional pastures [4,5].

The intestinal microbiota plays a vital role in ruminants’ digestion and nutrient absorption, including alpacas, influencing the breakdown of cellulose, hemicellulose, and other nutritional components [6,7]. Unlike true ruminants with four stomach chambers, alpacas have a three-chambered stomach, with the first compartment (C1) serving as the primary site for microbial fermentation. Structurally and functionally analogous to the rumen, the C1 occupies most of the stomach’s volume and is the main site for the microbial breakdown of fibrous plant material [8]. The microbial ecosystem of the C1 includes diverse bacteria, archaea, fungi, and protozoa, each with specific roles. Bacteria dominate in number and are essential for degrading plant polysaccharides like cellulose and hemicellulose [7]. Methanogenic archaea, representing about 90% of the archaeal population, contribute to methane production as a byproduct of fermentation [9,10]. Anaerobic fungi play a crucial role in breaking down lignified plant cell walls through enzymatic action [11,12]. Protozoa, while not essential, contribute to microbial population balance and fiber digestion [13,14]. These microbial interactions are critical for alpacas to thrive in nutrient-poor, high-altitude environments.

Metagenomics, particularly through the analysis of 16S and 18S rRNA genes, is a pivotal tool for studying these microbial communities. This approach provides detailed insights into the taxonomy, diversity, and ecological roles of the microorganisms present in the C1 [15,16,17]. Such analyses allow researchers to uncover how microbial populations adapt to environmental and dietary changes, offering a comprehensive understanding of the microbiota’s contribution to host health and adaptation [18].

Geographic location and diet are key factors influencing the composition of intestinal microbiota. Variations in altitude, climate, and vegetation affect the diversity and function of these microbial communities [19,20]. Changes in diet, whether abrupt or gradual, can directly alter microbial composition and may lead to metabolic disorders if the diet is inadequate [21,22]. Additionally, understanding the interactions between the microbiota and systemic physiological parameters, such as blood metabolites, provides crucial insights into host health and adaptation.

The interaction between the intestinal microbiota and blood parameters is essential for understanding the health and performance of ruminants, as these microbial communities influence not only digestion but also systemic processes such as immune modulation [23]. Recent studies have shown that changes in intestinal microbiota, induced by dietary variations, affect key blood metabolites, including short-chain fatty acids (SCFAs), triglycerides, and glucose, which play fundamental roles in metabolism and immune regulation [24,25]. SCFAs, in particular, act as energy sources for the host and modulate the immune response by influencing regulatory T cells and the production of pro-inflammatory cytokines [26,27]. Specific microorganisms, such as bacteria of the genus *Prevotella* and methanogenic archaea, may indirectly affect immune responses by altering metabolite production and maintaining ruminal homeostasis [28]. These relationships underscore how the C1 microbiota, including bacteria, fungi, and protozoa, interacts with the immune system and influences physiological parameters in the host. Evaluating these correlations is indispensable for understanding microbial adaptations in diverse environments, such as the Puna regions.

This study hypothesizes that the intestinal microbiota composition in alpacas differs between the two Puna regions due to their contrasting climates and diets. The objective is to evaluate these disparities and explore their implications for understanding microbial adaptations to environmental conditions.

## 2. Materials and Methods

### 2.1. Study Location and Weather Conditions

This study was conducted at the “La Raya” Experimental Center of the Universidad Nacional del Altiplano de Puno, located in Santa Rosa District, Melgar Province, Puno Department, Peru. The research focused on two agroecological regions, the Wet Puna and the Dry Puna, which differ significantly in climate, altitude, and vegetation.

The Wet Puna is situated at an elevation of 4200 m above sea level and experiences a semi-dry, cold climate with temperatures ranging from −12 °C to 19.75 °C and an annual rainfall of 932 mm [4]. The vegetation primarily consists of grasses such as *Stipa ichu*, *Festuca*, and *Calamagrostis*, which serve as the main food source for alpacas [29]. In contrast, the Dry Puna is located at 4900 m above sea level and is characterized by an arid, dry environment with temperatures ranging from −18 °C to 16 °C and an annual precipitation of 450–600 mm [4]. The predominant vegetation includes species like *Calamagrostis*, *Gentiana*, *Werneria*, and *Hypsela*, which form the core diet of alpacas in this region [29] (Table S1).

### 2.2. Sample Collection

The eight 18-month-old adult alpacas (Table 1) were free-range and managed in accordance with Peruvian National Legislation No. 30407, which pertains to Animal Protection and Welfare. These specimens were destined to be sacrificed. To collect samples, we retrieved the contents from the first stomach compartment (C1) of the alpaca’s digestive tract, obtaining around 500 g of material. Subsequently, this material underwent a sieving procedure with sterile gauze, resulting in the extraction of 20 mL of liquid. The liquid was then diluted at a 1:10 ratio using a 0.09% NaCl solution, yielding a total of 8 samples from compartment 1 of the digestive tract. The workflow of this study’s methodology is shown in Supplementary Figure S1.

### 2.3. Hematological Parameters

The hematological analysis (Table S1) involved evaluating both red and white blood cell series. The red blood cell count was performed using the hemocytometer method, in which blood samples were diluted with an isotonic solution to prevent agglutination and hemolysis and counted using a Neubauer chamber at 40× magnification. Hematocrit was determined using centrifugation, separating erythrocytes from plasma to measure the percentage of blood volume occupied by red blood cells. Hemoglobin concentration was measured spectrophotometrically using Drabkin’s reagent (Sigma, St. Louis, MO, USA), which converts hemoglobin to cyanmethemoglobin, with absorbance read at 540 nm. For the white blood cell count, samples were diluted in Turk’s solution to lyse erythrocytes, and counts were performed using a Neubauer chamber. The differential white blood cell count was conducted by preparing and staining a blood smear with Wright’s stain to identify and quantify different leukocyte types and observe any abnormalities [30].

### 2.4. DNA Extraction and Sequencing

Microbial DNA extraction was performed using the DNA Extract Plus DNA–QUANTABIO kit (Quantabio, Beverly, MA, USA), which was chosen for its efficiency in obtaining high-quality DNA suitable for metagenomic analyses. DNA quality was assessed using Qubit^®^ fluorometry (Thermo Fisher Scientific, Waltham, MA, USA) and 1% agarose gel electrophoresis to ensure accurate quantification and integrity. The V3–V4 region of the bacterial and archaeal 16S rRNA gene was amplified using primers 341F and 806R, following established protocols for optimal amplification of these conserved regions. Similarly, the V4 region of the 18S rRNA gene for protists and fungi was amplified using primers 528F and 706R, which were selected for their wide taxonomic coverage. Library preparation was conducted using the Illumina TruSeq^®^ DNA sample preparation kit (Illumina, San Diego, CA, USA), a widely used platform that ensures consistent and reliable sequencing outputs. Library quality was evaluated using the Qubit 2.0 fluorometer (Life Technologies, Carlsbad, CA, USA) and Agilent Bioanalyzer 2100 System (Agilent Technologies, Palo Alto, CA, USA), which provide precise quantification and fragment analysis critical for high-throughput sequencing.

### 2.5. Bioinformatics Analysis

The preparation and analysis of sequencing data were conducted using the Quantitative Insights Into Microbial Ecology (QIIME2) v2023.9 analytical platform [31], which was selected for its robust handling of large metagenomic datasets. Amplicon sequence variants (ASVs) were generated using the DADA2 protocol v.1.18 [32], which were chosen for their accuracy in denoising and resolving closely related variants. Taxonomic classification was performed using the QIIME2 inbuilt naïve Bayes classifier and calibrated with the Silva Reference database v.138.1, a comprehensive and frequently updated resource for 16S and 18S rRNA sequences. High-quality sequences were aligned with MAFFT v7 [33], which is known for its precision in multiple sequence alignment, and phylogenetic trees were constructed with the FastTree algorithm v2.1.11, which was selected for its efficiency in processing large datasets.

### 2.6. Statistical Analysis

Rarefaction curves were generated for each sample to ensure adequate sequencing depth with the minimum sample size. The alpha diversity of intestinal communities was assessed using Pielou, Shannon, and Simpson indices and calculated with the MicrobiotaProcess library [34], as these metrics provide complementary insights into microbial richness and evenness. The statistical analysis was conducted using the R package Phyloseq [35] in R (v4.1.1) [36], while group comparisons for alpha diversity employed the Kruskal–Wallis test, which was chosen for its suitability in analyzing non-parametric data. Beta diversity was determined using the Bray–Curtis dissimilarity index and visualized through a principal coordinate analysis (PCoA), and differences between groups were evaluated with PERMANOVA using 9999 permutations, which is a robust method for multivariate ecological data [37]. Differences in fungal and protist communities were further explored using LDA scores calculated with the LEfSe algorithm, which identifies taxa with significant differential abundances between groups. Taxa were displayed if LDA values > 2.0 and the *p* value was below 0.05.

## 3. Results

An analysis of variance (ANOVA) was performed to compare the hematological parameters of alpacas from the Central Andean Wet Puna (CAWP) and Central Andean Dry Puna (CADP) (Table 2). The evaluated variables included red blood cell count (RBC), hematocrit (HTO), hemoglobin concentration (HB), total white blood cell count (WBC), and the relative proportions of neutrophils (NEU%), lymphocytes (LINF%), monocytes (MON%), and eosinophils (EOS%). To explore post hoc differences between groups, Duncan’s multiple-range test was applied.

The results are presented as mean ± standard deviation for each group, with superscript letters indicating comparisons from the post hoc test. However, no statistically significant differences (*p* > 0.05) were found between the two groups in any of the parameters analyzed.

### 3.1. Alpha Diversity of Communities of Prokaryotic and Eukaryotes

To investigate variations in gut microbiota communities of bacteria (Figure S2), archaea (Figure S3), fungi (Figure S4), and protists (Figure S5) in relation to diet types, rarefaction curves were employed, confirming the dataset’s suitability for additional analysis.

**Figure 1:**
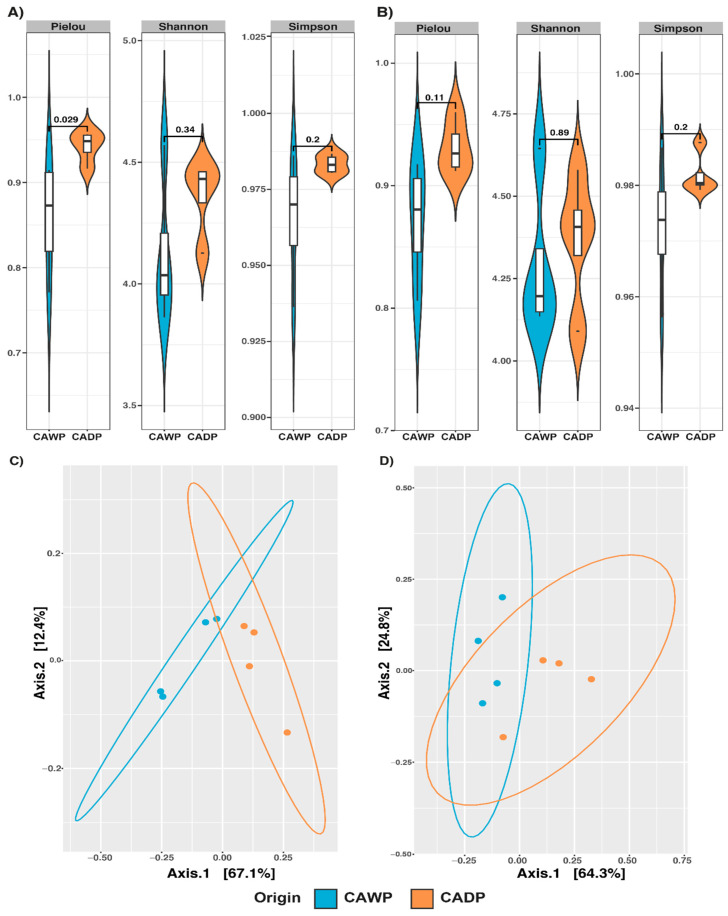
Alpha diversity Pielou, Shannon, and Simpson and principal coordinate analysis (PCoA) plot of beta diversity based on weighted UniFrac distance derived from sequencing data on microbiota between Central Andean Dry Puna and Central Andean Wet Puna groups. (**A**) Bacterial alpha diversity microbiota index. (**B**) Microbiota index of archaea alpha diversity. (**C**) Beta diversity of bacterial (**D**) Beta diversity of archaea. CADP = Central Andean Dry Puna, CAWP = Central Andean Wet Puna.

The results of the alpha diversity indices are presented in Figure 1. In the bacteria group (Figure 1A), a significant difference in the Pielou index (*p* = 0.029) was identified between the Wet Puna and Dry Puna groups, indicating greater evenness in the bacterial community of the Wet Puna. In the archaea group (Figure 1B), although no statistically significant differences were observed, an increase in alpha diversity was noted in the Wet Puna region, suggesting a potential trend linked to environmental conditions.

**Figure 2:**
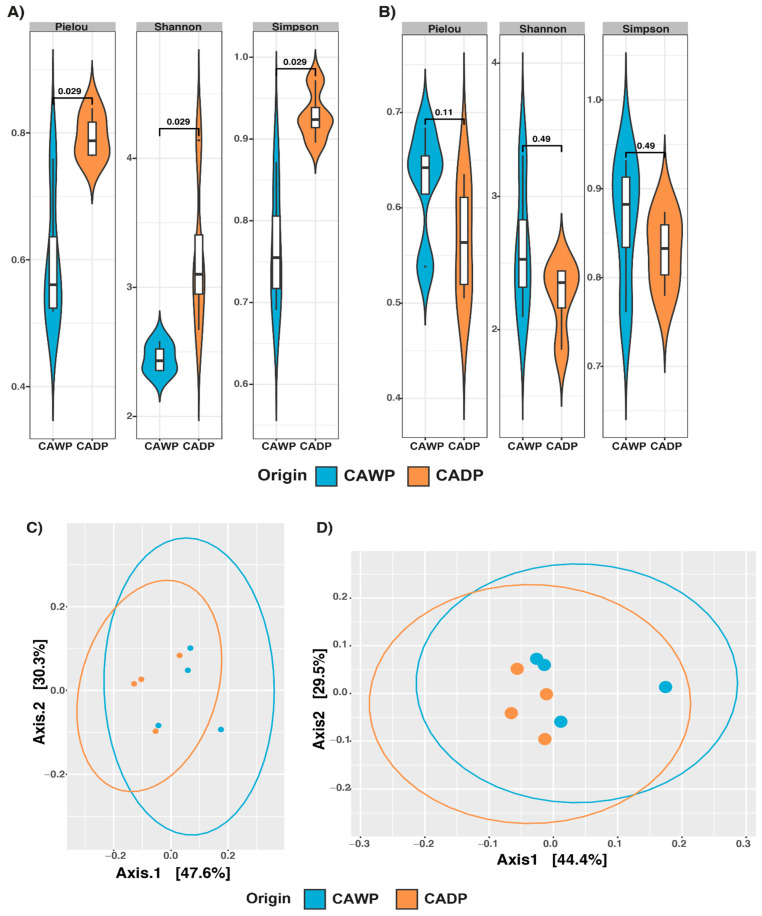
Alpha diversity Pielou, Shannon, and Simpson and principal coordinate analysis (PCoA) plot of beta diversity based on weighted UniFrac distance derived from sequencing data on microbiota between Central Andean Dry Puna and Central Andean Wet Puna groups. (**A**) Fungi alpha diversity microbiota index. (**B**) Microbiota index of protist alpha diversity. (**C**) Beta diversity of fungi (**D**) Beta diversity of protists. CADP = Central Andean Dry Puna, CAWP = Central Andean Wet Puna.

For fungi (Figure 2A), significant differences were identified across multiple indices: Pielou (*p* = 0.029), Shannon (*p* = 0.029), and Simpson (*p* = 0.029). These findings highlight greater richness and diversity in fungal communities in the Wet Puna, potentially reflecting variations in diet and environmental conditions. In contrast, while the protists group (Figure 2B) did not present statistically significant differences in composition, a notable increase in alpha diversity was recorded in the Wet Puna, indicating a higher richness in this region.

### 3.2. Composition of Bacteria and Eukaryote Communities

A beta diversity analysis was performed using the weighted UniFrac distance to evaluate differences in microbiota composition between the Dry Puna and Wet Puna groups. The results, which were visualized using a principal coordinate analysis (PCoA) (Figure 2), highlight notable trends in microbial community divergence.

A PERMANOVA analysis (Table 3) validated these findings. In the bacteria group (Figure 1C), a significant divergence (*p* = 0.0284) was detected, with distinct clustering between the diet groups. For archaea (Figure 1D), dispersion between groups was significant (*p* = 0.0287), suggesting diet-associated differences in archaeal communities. Conversely, no significant dispersion or clustering was identified in the fungi (Figure 2C; *p* = 0.2021) or protists (Figure 2D; *p* = 0.9138) groups, indicating the minimal impact of diet on these communities at the compositional level.

**Figure 3:**
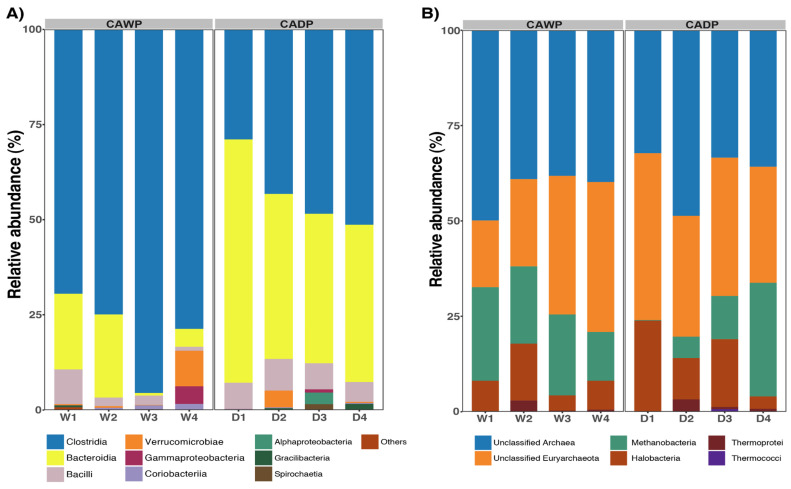
Relative abundances were observed in CAWP and CADP groups. Only the most represented taxa are presented. (**A**) Relative abundance of the most predominant classes in bacteria. (**B**) Relative abundance of the most abundant classes in archaea. CADP = Central Andean Dry Puna, CAWP = Central Andean Wet Puna.

In the bacteria group (Figure 3A), notable differences in taxonomic composition were identified between the Wet and Dry Puna groups. Clostridia was the predominant class in the Wet Puna, accounting for 79% of the community, compared to 43% in the Dry Puna. Conversely, Bacteroidia was more abundant in the Dry Puna (49%) than in the Wet Puna (12%). Both Bacilli and Verrucomicrobiae were consistently represented at 5% in each group. Additionally, Gammaproteobacteria accounted for 4% in the Wet Puna and 1% in the Dry Puna. Minor classes, including Coriobacteriia, Alphaproteobacteria, Gracillibacteria, and Spirochaetia, each represented 1% in both regions.

In the archaea group (Figure 3B), the unclassified class was the largest group, representing 41% in the Wet Puna and 38% in the Dry Puna. Similarly, the unclassified Euryarchaeota accounted for 29% in the Wet Puna and 34.39% in the Dry Puna. Methanobacteria was more prevalent in the Wet Puna (20%) than in the Dry Puna (12%), while Halobacteria showed the opposite trend, with 15% in the Dry Puna and 8% in the Wet Puna. Thermoprotei and Thermococci each contributed 1% to the composition in both groups.

**Figure 4:**
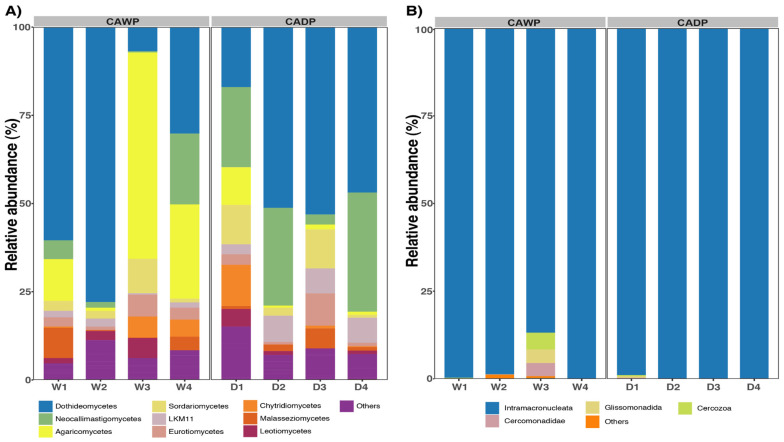
Relative abundances were observed in CAWP and CADP groups. Only the most represented taxa are presented. (**A**) Relative abundance of the most predominant classes in fungi. (**B**) Relative abundance of the most abundant classes in protists. CADP = Central Andean Dry Puna, CAWP = Central Andean Wet Puna.

In the fungi group (Figure 4A), a higher variability in composition was observed. Dothideomycetes dominated in both regions, accounting for 42% in the Wet Puna and 43% in the Dry Puna. Neocallimastigomycetes were more abundant in the Dry Puna (20.8%) than in the Wet Puna (6%). Conversely, Agaricomycetes were more prominent in the Wet Puna (24%) compared to the Dry Puna (3.37%). Sordariomycetes represented 4% in the Wet Puna and 6% in the Dry Puna, while the remaining classes collectively accounted for 5%.

In the protists group (Figure 4B), Intramacronucleata was overwhelmingly dominant in both regions, constituting 96% of the community in the Wet Puna and 99.7% in the Dry Puna. In the Dry Puna, additional classes were identified, although each accounted for less than 5% of the community: uncultured Cercozoa (4%), Glissomonadida (3.9%), and Cercomonadidae (3.7%). Other classes collectively represented 1%.

### 3.3. Identification of Biomarkers Associated with Procedence

A linear discriminant analysis (LDA) was applied to evaluate the effect size and characterize distinctions in the microbiota composition of compartment 1 between alpacas inhabiting the Wet and Dry Puna regions.

**Figure 5:**
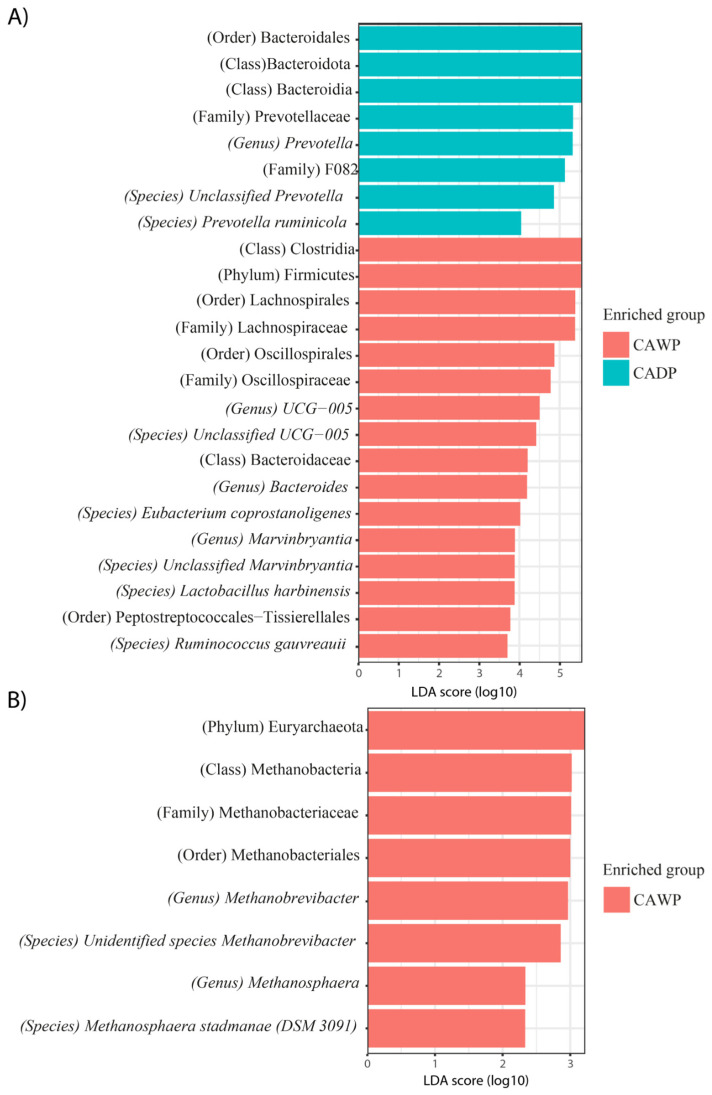
Differences in the intestinal bacterial and archaea microbiota between CAWP and CADP alpaca groups. Bar chart of linear discriminant analysis (LDA) for differentially abundant genera: (**A**) Bacteria in CAWP versus CADP; (**B**) Archaea only in CAWP. CADP = Central Andean Dry Puna, CAWP = Central Andean Wet Puna.

In Figure 5A, the Wet Puna group is enriched with bacterial taxa, such as Bacteroidales, Bacteroidota, Bacteroidia, Prevotellaceae, *Prevotella*, *Prevotella ruminicola*, and unidentified Prevotella species. The Dry Puna group is characterized by taxa including Clostridia, Firmicutes, Lachnospirales, Lachnospiraceae, Oscillospirales, Oscillospiraceae, Ruminococcaceae *UCG-005*, unidentified species *UCG-005*, Bacteroidaceae, *Bacteroides*, *Eubacterium coprostanoligenes*, *Marvinbryantia*, and unidentified *Marvinbryantia* species, *Lactobacillus harbinensis*, *Peptostreptococcales-Tissierellales*, and *Ruminococcus gauvreauii*.

In Figure 5B, archaea are observed exclusively in the Wet Puna group, represented by taxa such as Euryarchaeota, *Methanobacteria*, *Methanobacteriaceae*, *Methanobacteriales*, *Methanobrevibacter*, unidentified *Methanobrevibacter* species, *Methanosphaera*, and *Methanosphaera stadtmanae* (DSM 3091).

**Figure 6:**
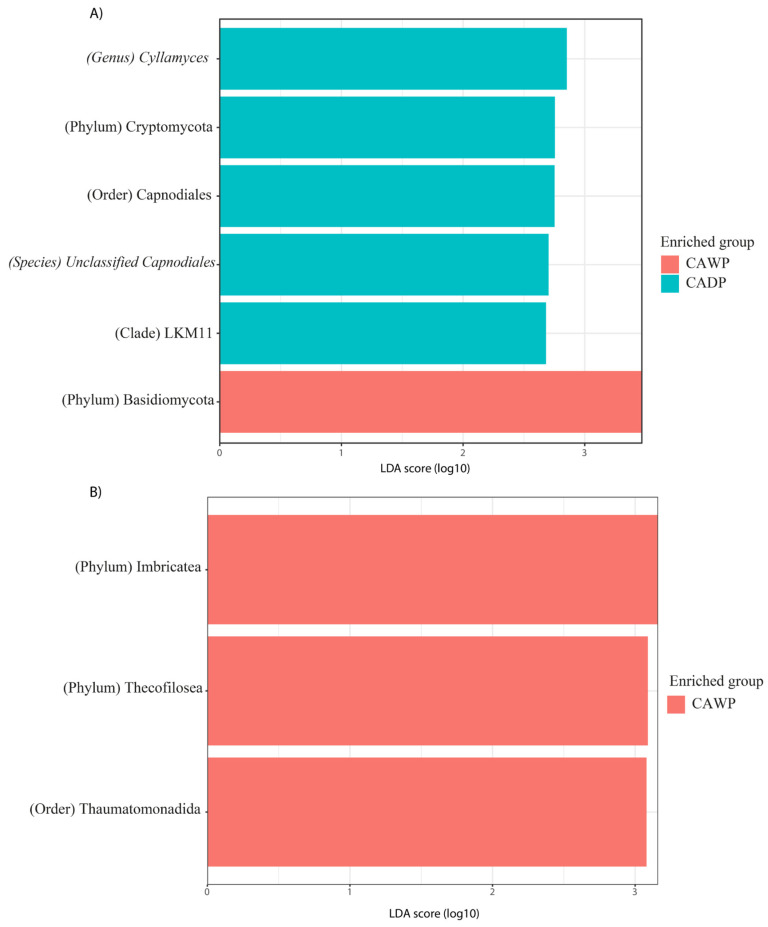
Differences in the intestinal fungi and protist microbiota between CAWP and CADP alpaca groups. Bar chart of linear discriminant analysis (LDA) for differentially abundant genera: (**A**) Fungi in CAWP versus CADP; (**B**) Protists only in CAWP. CADP = Central Andean Dry Puna, CAWP = Central Andean Wet Puna.

In Figure 6A, fungal taxa in the Wet Puna group include Cyllamyces, Cryptomycota, Capnodiales, unidentified *Capnodiales* species, and LKM11, while the Dry Puna group is represented by Basidiomycota.

Finally, in Figure 6B, protists are exclusively identified in the Dry Puna group, including Imbricatea, Thecofilosea, and Thaumatomonadida.

### 3.4. Microbial Community Structure Dynamics

**Figure 7:**
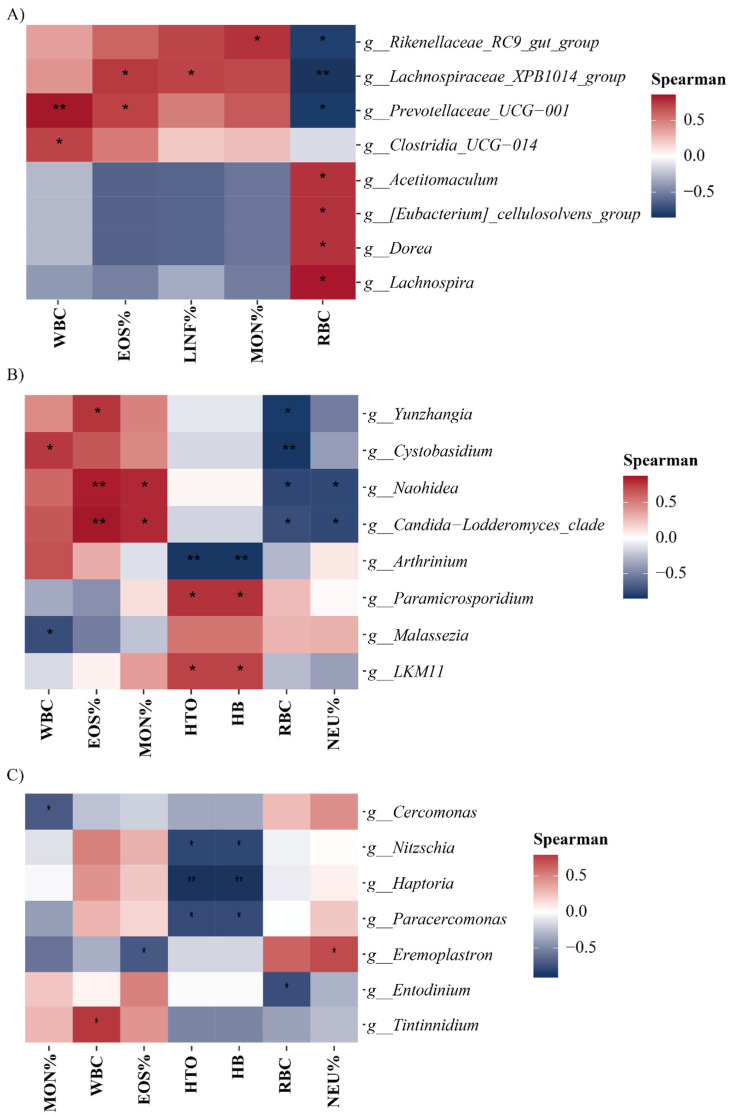
Spearman correlation heatmap between intestinal microbial genera and hematological variables. * *p* < 0.05; ** *p* < 0.01. (**A**) Heatmap for bacterial genera, (**B**) heatmap for fungal genera, and (**C**) heatmap for protist genera.

A Spearman correlation analysis (Figure 7) revealed associations between the relative abundance of microorganisms and hematological parameters in alpacas. In bacteria, WBC exhibited a significant positive correlation with *Prevotellaceae_UCG-001* and *Clostridia_UCG-014*. EOS% was significantly correlated with *Lachnospiraceae_XPB1014*_group and *Prevotellaceae_UCG-001*, while LINF% had a significant positive correlation with *Lachnospiraceae_XPB1014*_group. MON% displayed a significant positive correlation with *Rikenellaceae_RC9*_gut_group. RBC was positively correlated with *Acetitomaculum, Eubacterium_cellulosolvens*_group, *Dorea*, and *Lachnospira*, and negatively correlated with *Rikenellaceae_RC9*_gut_group, *Lachnospiraceae_XPB1014*_group, and *Prevotellaceae_UCG-001*.

In fungi, WBC had a significant negative correlation with *Malassezia* and a positive correlation with *Cystobasidium*. EOS% was positively correlated with *Yunzhangia*. Similarly, MON% had a significant positive correlation with *Naohidea* and *Candida-Lodderomyces*_clade. HTO and HB were positively correlated with *LKM11* and *Paramicrosporidium* and negatively correlated with *Arthrinium*. RBC exhibited a significant negative correlation with *Yunzhangia* and *Cystobasidium*, whereas NEU% had a significant negative correlation with *Naohidea* and *Candida-Lodderomyces*_clade.

In protists, MON% had a significant negative correlation with *Cercomonas*, while WBC was positively correlated with *Tintinnidium*. EOS% displayed a significant negative correlation with *Eremoplastron*. HTO and HB were negatively correlated with *Nitzschia*, *Haptoria*, and *Paracercomonas*. RBC had a significant negative correlation with *Entodinium*, and NEU% was positively correlated with *Eremoplastron*.

A canonical correspondence analysis (CCA) (Figure S6) was performed to explore the associations between microbial composition and hematological parameters, differentiated by origin. For bacteria (Figure S6A), CADP was primarily associated with the hematological parameters MON% and LINF%, while CAWP exhibited relationships with *Flavonifractor*, UCG-008, and *Pediococcus*. In the case of archaea (Figure S6B), a clear separation between CAWP and CADP was observed, in which CAWP was linked to *Methanosphaera* and variables such as EOS%, LINF%, and MON%, whereas CADP was correlated with *Methanobrevibacter*, *Methanobacterium*, and the parameters NEU%, RBC, and HTO. For fungi (Figure S6C), no clear separation between the groups was detected, but trends indicated that CAWP was associated with MON%, EOS%, WBC, and LINF%, along with the genera *Wallemia*, *Papiliotrema*, *Mucor*, *Konldoa*, and *Suillus*, while CADP was correlated with NEU%, RBC, HTO, and HB. Finally, for protists (Figure S6D), a central overlap was observed between the groups, suggesting little differentiation; however, CAWP was associated with EOS%, WBC, LINF%, and MON%, along with *Chaetoceros*, *Conidiobolus*, *Cyclotella*, and *Katablepharis*, while CADP was correlated with the hematological parameters NEU%, RBC, HB, and HTO.

## 4. Discussion

This study aimed to explore disparities in the community composition of anaerobic fungi, bacteria, archaea, and protists in compartment 1 of alpacas with regard to diet. The findings reveal significant differences in the intestinal microbiota between the contrasting environments of the Wet and Dry Puna regions. These results provide a deeper understanding of the taxonomic composition, richness, and structure of the microbiota, shedding light on the relationship between diet and microbial diversity.

The Wet Puna region exhibited greater alpha diversity, particularly in bacteria and fungi, while no significant differences were identified in archaea and protists, although a trend of higher richness was observed in protists in the Wet Puna. This aligns with previous research on ruminants, in which diet and environmental conditions have been shown to influence microbial richness, particularly bacterial communities, which are more responsive to dietary and host-related factors [11]. For fungi, the observed increased richness in the Wet Puna region is consistent with findings in cows [38], suggesting that fungal diversity may vary with seasonality, diet, and environmental conditions. No significant differences were observed in fungal composition between the regions; however, studies in cows have reported structural changes in anaerobic fungal communities influenced by diet [38]. It is essential to consider that these observations might be influenced by limitations inherent to the study, such as the limited number of animals per group.

Significant variations in bacterial composition between the Wet and Dry Puna regions were identified, which is a trend that has also been documented in other ruminants [11]. Previous studies have indicated that microbial communities in ruminants differ depending on diet and host factors, with beta diversity being strongly influenced by these variables [11]. The taxonomic composition of bacterial communities in this study aligns with the existing literature, highlighting the prevalence of Clostridia, Bacteroidia, and Bacilli in ruminants such as cattle [39,40], camels [41], and goats [42,43]. In archaea, the predominant taxa included unidentified classes of Euryarchaeota, Methanobacteria, and Halobacteria, which is consistent with findings in alpacas [44], cows [45,46], and camels [47]. Regarding fungi, the most prevalent classes identified were Dothideomycetes, Neocallimastigomycetes, Agaricomycetes, and Sordariomycetes, aligning with studies in primates [48], goats [49], and cows [50]. In protists, Intramacronucleata was predominant, as previously documented in cows [51]. However, the limited research on protist microbiota in ruminants restricts further classification and understanding of these groups.

The identification of specific microbial taxa associated with dietary environments provides valuable insights. In the Wet Puna, *Prevotella ruminicola* was prominent, highlighting the genus’s saccharolytic activity [52] and its role in generating short-chain fatty acids (SCFAs) [53], which is essential for hepatic gluconeogenesis under glucose-limited conditions [54]. *Prevotella* also modulates hydrogen flux, potentially mitigating methane production [55,56], which is a critical adaptation for energy efficiency in resource-scarce environments. These findings underscore the metabolic versatility of alpacas, enabling them to maximize energy extraction from available resources in harsh conditions like the Dry Puna.

In the Wet Puna group, the presence of *Lactobacillus harbinensis* suggests a potential correlation between this beneficial bacterial genus and the adaptability of alpacas to humid conditions. *Lactobacillus* is associated with host health and is generally considered beneficial [57]. Further research is needed to clarify its specific role in alpacas and its contribution to adaptation in the Wet Puna. Additionally, *Eubacterium coprostanoligenes* was identified in the Wet Puna group, likely influenced by the fermentation of dietary fibers characteristic of the region’s vegetation [58,59]. This genus’s ability to adapt to fiber-rich diets highlights its importance in modulating alpaca gut microbiota under varying dietary conditions.

In the Dry Puna, *Methanobrevibacter* and *Methanosphaera* were identified, which is consistent with their known roles in methane production via hydrogen and carbon dioxide utilization [60]. These methanogens enhance fiber degradation [61], which is a critical adaptation for efficient nutrient utilization in arid environments. Previous studies have also indicated that diet variation influences the prevalence of archaeal genera, as observed in goats [62]. The fungal genus *Cyllamyces*, identified as a biomarker in the Wet Puna, has been previously reported in dromedary camels [63], cows, and buffaloes [64,65]. Its abundance is correlated with the nutritional quality of forage and seasonal variations, highlighting the significant influence of diet on anaerobic fungal communities [66].

The significant positive correlation between *Acetitomaculum* and red blood cells (RBC) in alpacas suggests a relationship between the metabolic activity of this bacterium and the host’s energy metabolism. *Acetitomaculum*, a member of the Lachnospiraceae family, is known for its ability to ferment complex carbohydrates and dietary polysaccharides, producing short-chain fatty acids (SCFAs) such as acetate, butyrate, and propionate, which are key energy sources for the ruminal epithelium [25,67]. These SCFAs not only contribute to maintaining gastrointestinal epithelial integrity but also facilitate the absorption of essential nutrients such as iron, folic acid, and vitamin B12, which are critical for hemoglobin synthesis and red blood cell formation [68]. Furthermore, previous studies have demonstrated that Acetitomaculum abundance increases in diets rich in fermentable carbohydrates, suggesting enhanced ruminal metabolic efficiency and improved nutrient availability [67]. In this context, the greater abundance of *Acetitomaculum* observed in the present study could be linked to better energy and nutrient availability, which is reflected in the observed increase in RBC. These findings highlight the potential functional role of *Acetitomaculum* in alpacas’ hematological health and suggest the need for further studies to better understand its specific contribution to ruminal metabolism and its impact on the overall well-being of the host.

The significant negative correlation between *Malassezia* and total white blood cell count (WBC) in alpacas suggests a potential interaction between this fungus and the host’s systemic immune response. While *Malassezia* is primarily known as a component of the skin microbiota, recent studies have demonstrated its ability to colonize the gastrointestinal tract, even under low oxygen and high-temperature conditions [69]. In humans, its presence in the gut microbiota has been associated with both healthy and pathological states, showing potential immunomodulatory effects, particularly in inflammatory processes [70]. The observed negative correlation in alpacas may indicate that higher *Malassezia* abundance is associated with a downregulation of systemic immune responses, possibly reducing the stimulus for leukocyte proliferation. Alternatively, this relationship could reflect a host adaptation to the presence of *Malassezia*, minimizing excessive immune activation. Further research is needed to determine whether *Malassezia* functions as an immune modulator in alpacas or if its abundance reflects changes in the intestinal environment that influence the host’s immune status.

The significant positive correlation between *Eremoplastron* and NEU% in alpacas may suggest an interaction between the presence of this ciliate protozoan and the host’s innate immune response. *Eremoplastron* has been reported to be more abundant in immature ruminal microbiomes or in young animals, where its predominance might be linked to increased production of soluble metabolites, such as volatile fatty acids or proteolytic byproducts, that could act as signals for neutrophil activation [71,72]. Additionally, previous studies have associated *Eremoplastron* with animals exhibiting lower feed efficiency and less metabolically efficient rumens [73], potentially creating a metabolically demanding environment prone to neutrophil-mediated inflammatory responses. In this context, increased neutrophil counts (NEU%) may reflect an adaptive mechanism of the host’s immune system in response to microbial activity or specific metabolites derived from *Eremoplastron*. However, this association might also indicate shifts in ruminal microbiome homeostasis during developmental stages or periods of metabolic stress, emphasizing the need for further studies to elucidate the functional role of *Eremoplastron* in modulating local immune responses in the rumen of alpacas.

The association of the CADP group with elevated levels of MON%, LINF%, and EOS%, which was identified through the canonical correspondence analysis (CCA), suggests specific immunological adaptations of alpacas to the extreme conditions of the Dry Puna, characterized by chronic hypoxia and low temperatures. Increased MON% may indicate enhanced tissue surveillance and repair mechanisms driven by growth factors induced under hypoxic stress, while elevated LINF% reflects efficient adaptive immune responses mediated by cytokines targeting local pathogens [74,75]. Similarly, higher EOS% levels may reflect immune responses to parasites or allergens specific to the habitat [74]. Additionally, the intestinal microbiota, which is shaped by environmental and dietary factors, interacts with the host immune system through metabolites such as short-chain fatty acids, promoting immune cell differentiation and function [76]. These findings underscore the role of host–microbiota interactions in the immunological resilience of alpacas in high-altitude ecosystems.

Likewise, the association of the CAWP group with microbial genera such as *Flavonifractor*, *UCG-008*, *Methanobrevibacter*, and *Methanobacterium*, which was revealed by the CCA, highlights microbial adaptations that are critical for optimizing metabolic efficiency and resilience in high-altitude environments. Genera like *Flavonifractor* and *UCG-008*, which are involved in polysaccharide degradation and short-chain fatty acid production, likely enhance energy extraction from a fiber-rich diet [77,78]. Concurrently, methanogenic archaea, such as *Methanobrevibacter* and *Methanobacterium*, contribute to ruminal fermentation balance and pH stability, which are essential for nutrient utilization [79,80]. These results emphasize the symbiotic relationship between the host and microbiota, in which microbial ecosystems not only support the dietary and physiological needs of alpacas but also play a crucial role in their adaptation and productivity in challenging high-altitude habitats.

The findings of this study have important implications for the husbandry, management, and conservation of alpacas in high-altitude environments. Identifying beneficial microbes, such as *Flavonifractor* and *UCG-008*, highlights the potential for targeted nutritional strategies to enhance metabolic efficiency, while monitoring hematological variables (MON%, LINF%, EOS%) can provide insights into alpaca health under environmental or dietary stress. Preserving the ecological niches of the Wet and Dry Puna regions is crucial, as these habitats shape the microbial and physiological adaptations that support alpaca resilience, emphasizing the importance of sustainable grazing practices and habitat conservation.

## 5. Conclusions

This study reveals significant differences in the intestinal microbiota of alpacas between the Wet and Dry Puna regions, highlighting the influence of environmental and dietary factors on microbial diversity and composition. Key microbial taxa, such as *Prevotella, Eubacterium coprostanoligenes*, and *Methanobrevibacter*, were identified, demonstrating their potential roles in fiber degradation, energy metabolism, and adaptation to high-altitude conditions. Correlations between specific microbial genera and hematological variables, such as *Acetitomaculum* with RBC and *Eremoplastron* with NEU%, provide insights into host–microbiota interactions that support alpaca resilience in challenging environments. These findings have practical implications for alpaca husbandry and management, suggesting the potential for targeted dietary strategies to enhance metabolic efficiency and health. Additionally, preserving the natural habitats of the Wet and Dry Puna is essential to maintain the unique microbial adaptations that underpin alpaca productivity. Future research should explore the functional roles of these microbial communities and their applications in sustainable livestock management.

## Figures and Tables

**Table 1:** Characteristics of alpaca samples from Central Andean Dry and Wet Puna.

Origin	Altitude	Diet	Sample ID	Sex	Age (Month)
Central Andean dry Puna	4200 m above sea level	*Stipa ichu-Festuca-Calamagrostis*	D1	Male	18
			D2	Male	18
			D3	Male	18
			D4	Male	18
Central Andean wet Puna	4900 m above sea level	*Carex-Gentiana-Werneria-Arenaria-Hypsela*	W1	Male	18
			W2	Male	18
			W3	Male	18
			W4	Male	18

**Table 2:** Hematological parameters of alpacas from Central Andean Dry and Wet Puna.

Origin	RBC	HTO	HB	WBC	NEU%	LINF%	MON%	EOS%
CADP	8.55 ± 1.9 a	34 ± 4.69 a	13.6 ± 1.88 a	11.31 ± 2.38 a	48 ± 2.94 a	41.5 ± 1.73 a	6 ± 0.82 a	4.25 ± 0.5 a
CAWP	10.57 ± 2.31 a	31.75 ± 3.5 a	12.7 ± 1.4 a	11.18 ± 3.99 a	49.5 ± 3.32 a	40.5 ± 1.73 a	5.5 ± 1 a	4.25 ± 1.26 a

Group means (±standard deviation) within the same columns followed by the same letter are not significantly different (*p* < 0.05) according to Duncan’s test. CADP = Central Andean Dry Puna, CAWP = Central Andean Wet Puna, RBC = erythrocytes, HTO = hematocrit, HB = hemoglobin, WBC = white blood cell count, NEU% = neutrophil percentage, LINF% = percentage of lymphocytes, MON% = monocyte percentage, EOS% = percentage of eosinophils.

**Table 3:** Two-way PERMANOVA of the weighted UniFrac distance regarding the Procedence of the alpacas.

Items	Df	SumOfSqs	R2	F	Pr(>F)
Procedence					
Bacteria	1	0.16134	0.51019	6.2496	0.0284 *
Archae	1	0.14832	0.5128	6.3154	0.0287 *
Fungi	1	0.030352	0.18975	1.4051	0.2021
Protist	1	0.006719	7439	0.4822	0.9138

Signif. codes: * *p* < 0.05.

## Data Availability

The original contributions presented in the study are included in the article/Supplementary Materials, further inquiries can be directed to the corresponding authors.

## Supplementary Materials

The following supporting information can be downloaded at https://www.mdpi.com/article/10.3390/microorganisms14010138/s1: Figure S1. Study flowchart. Figure S2. Species richness rarefaction curves show sequencing depth of 16S data obtained from bacteria from compartment 1 samples. Figure S3. Species richness rarefaction curves show sequencing depth of 16S data obtained from archaea from compartment 1 samples. Figure S4. Species richness rarefaction curves show sequencing depth of 18S data obtained from fungi from compartment 1 samples. Figure S5. Species richness rarefaction curves show sequencing depth of 18S data obtained from protists from compartment 1 samples. Figure S6: Canonical correlation analysis for origin. Table S1. Detailed environmental and climatic conditions of study locations.

## References

[1] Rojas M., Manchego A., Rocha C.B., Fornells L.A., Silva R.C., Mendes G.S., Dias H.G., Sandoval N., Pezo D., Santos N. (2016). Outbreak of Diarrhea among Preweaning Alpacas (*Vicugna pacos*) in the Southern Peruvian Highland. J. Infect. Dev. Ctries..

[2] Vásquez O.R., Gómez-Quispe O.E., Quispe P.E. (2015). Características Tecnológicas de La Fibra Blanca de Alpaca Huacaya En La Zona Altoandina de Apurímac: Technological Characteristics of the White Fibre of Huacaya Alpaca in Theandean Region of Apurimac. Rev. Investig. Vet. Perú.

[3] Bustinza Choque A.V., Machaca Machaca V., Cano Fuentes V., Quispe Coaquira J., Bustinza Choque A.V., Machaca Machaca V., Cano Fuentes V., Quispe Coaquira J. (2021). Evolución y Desarrollo de Las Razas de Alpaca: Suri y Huacaya. Rev. Investig. Vet. Perú.

[4] Paredes M., Condemayta C., Charaja C. (2009). Causas de mortalidad de alpacas en tres principales centros de producción ubicados en puna seca y humeda del departamento de Puno (Causes of mortality of alpacas in three main centers of production located in dry and humid fist of the Puno department). Rev. Electrónica Vet..

[5] Gómez-Quispe O.E., Rodríguez E.L., Benites R.M., Valenzuela S., Moscoso-Muñoz J., Ibañez V., Youngs C.R. (2022). Analysis of Alpaca (*Vicugna pacos*) Cria Survival under Extensive Management Conditions in the High Elevations of the Andes Mountains of Peru. Small Rumin. Res..

[6] Belanche A., Patra A.K., Morgavi D.P., Suen G., Newbold C.J., Yáñez-Ruiz D.R. (2021). Editorial: Gut Microbiome Modulation in Ruminants: Enhancing Advantages and Minimizing Drawbacks. Front. Microbiol..

[7] Jewell K.A., McCormick C.A., Odt C.L., Weimer P.J., Suen G. (2015). Ruminal Bacterial Community Composition in Dairy Cows Is Dynamic over the Course of Two Lactations and Correlates with Feed Efficiency. Appl. Environ. Microbiol..

[8] Weimer P.J. (2022). Degradation of Cellulose and Hemicellulose by Ruminal Microorganisms. Microorganisms.

[9] Efenberger M., Brzezińska-Błaszczyk E., Wódz K. (2014). Archaeons-still unknown microorganisms. Postępy Hig. Med. Dośw..

[10] Kassow A., Blank B., Paulsen H.M., Aulrich K., Rahmann G. (2010). Studies on Greenhouse Gas Emissions in Organic and Conventional Dairy Farms. Ressortforschung für den Ökologischen Landbau 2009.

[11] Henderson G., Cox F., Ganesh S., Jonker A., Young W., Janssen P.H. (2015). Rumen Microbial Community Composition Varies with Diet and Host, but a Core Microbiome Is Found across a Wide Geographical Range. Sci. Rep..

[12] De Nardi R., Marchesini G., Li S., Khafipour E., Plaizier K.J.C., Gianesella M., Ricci R., Andrighetto I., Segato S. (2016). Metagenomic Analysis of Rumen Microbial Population in Dairy Heifers Fed a High Grain Diet Supplemented with Dicarboxylic Acids or Polyphenols. BMC Vet. Res..

[13] Vďačný P., Orsi W., Bourland W.A., Shimano S., Epstein S.S., Foissner W. (2011). Morphological and Molecular Phylogeny of Dileptid and Tracheliid Ciliates: Resolution at the Base of the Class Litostomatea (Ciliophora, Rhynchostomatia). Eur. J. Protistol..

[14] Newbold C.J., de la Fuente G., Belanche A., Ramos-Morales E., McEwan N.R. (2015). The Role of Ciliate Protozoa in the Rumen. Front. Microbiol..

[15] Kim H.B., Isaacson R.E. (2015). The Pig Gut Microbial Diversity: Understanding the Pig Gut Microbial Ecology through the next Generation High Throughput Sequencing. Vet. Microbiol..

[16] Kim M., Park T., Yu Z. (2017). Metagenomic Investigation of Gastrointestinal Microbiome in Cattle. Asian-Australas. J. Anim. Sci..

[17] Hadziavdic K., Lekang K., Lanzen A., Jonassen I., Thompson E.M., Troedsson C. (2014). Characterization of the 18S rRNA Gene for Designing Universal Eukaryote Specific Primers. PLoS ONE.

[18] Qi S., Wang J., Zhang Y., Naz M., Afzal M.R., Du D., Dai Z. (2023). Omics Approaches in Invasion Biology: Understanding Mechanisms and Impacts on Ecological Health. Plants.

[19] Yatsunenko T., Rey F.E., Manary M.J., Trehan I., Dominguez-Bello M.G., Contreras M., Magris M., Hidalgo G., Baldassano R.N., Anokhin A.P. (2012). Human Gut Microbiome Viewed across Age and Geography. Nature.

[20] Linnenbrink M., Wang J., Hardouin E.A., Künzel S., Metzler D., Baines J.F. (2013). The Role of Biogeography in Shaping Diversity of the Intestinal Microbiota in House Mice. Mol. Ecol..

[21] McCann J.C., Wickersham T.A., Loor J.J. (2014). High-Throughput Methods Redefine the Rumen Microbiome and Its Relationship with Nutrition and Metabolism. Bioinforma. Biol. Insights.

[22] Khafipour E., Li S., Plaizier J.C., Krause D.O. (2009). Rumen Microbiome Composition Determined Using Two Nutritional Models of Subacute Ruminal Acidosis. Appl. Environ. Microbiol..

[23] Negash A. (2022). Gut Microbiota Ecology Role in Animal Nutrition and Health Performance. J. Clin. Microbiol. Antimicrob..

[24] He Z., Dong H. (2023). The Roles of Short-Chain Fatty Acids Derived from Colonic Bacteria Fermentation of Non-Digestible Carbohydrates and Exogenous Forms in Ameliorating Intestinal Mucosal Immunity of Young Ruminants. Front. Immunol..

[25] Shen Y., Jiang Y., Zhang S., Zou J., Gao X., Song Y., Zhang Y., Hu Y., Huang Y., Jiang Q. (2022). The Effect of Dietary Supplementation with Resveratrol on Growth Performance, Carcass and Meat Quality, Blood Lipid Levels and Ruminal Microbiota in Fattening Goats. Foods.

[26] Rooks M.G., Garrett W.S. (2016). Gut Microbiota, Metabolites and Host Immunity. Nat. Rev. Immunol..

[27] Shibata N., Kunisawa J., Kiyono H. (2017). Dietary and Microbial Metabolites in the Regulation of Host Immunity. Front. Microbiol..

[28] Leng R.A. (2018). Unravelling Methanogenesis in Ruminants, Horses and Kangaroos: The Links between Gut Anatomy, Microbial Biofilms and Host Immunity. Anim. Prod. Sci..

[29] Huanca Mamani T., Apaza Castillo N., Sapana Valdivia R. (2007). Defectos Congénitos y Hereditarios Visibles en Alpacas de dos Zonas Representativas de la Región Puno.

[30] Latimer K.S. (2011). Duncan and Prasse’s Veterinary Laboratory Medicine: Clinical Pathology.

[31] Bolyen E., Rideout J.R., Dillon M.R., Bokulich N.A., Abnet C.C., Al-Ghalith G.A., Alexander H., Alm E.J., Arumugam M., Asnicar F. (2019). Reproducible, Interactive, Scalable and Extensible Microbiome Data Science Using QIIME 2. Nat. Biotechnol..

[32] Callahan B.J., McMurdie P.J., Rosen M.J., Han A.W., Johnson A.J.A., Holmes S.P. (2016). DADA2: High-Resolution Sample Inference from Illumina Amplicon Data. Nat. Methods.

[33] Katoh K., Misawa K., Kuma K., Miyata T. (2002). MAFFT: A Novel Method for Rapid Multiple Sequence Alignment Based on Fast Fourier Transform. Nucleic Acids Res..

[34] Xu S., Zhan L., Tang W., Wang Q., Dai Z., Zhou L., Feng T., Chen M., Wu T., Hu E. (2023). MicrobiotaProcess: A Comprehensive R Package for Deep Mining Microbiome. Innovation.

[35] McMurdie P.J., Holmes S. (2013). Phyloseq: An R Package for Reproducible Interactive Analysis and Graphics of Microbiome Census Data. PLoS ONE.

[36] R Core Team (2020). R: A Language and Environment for Statistical Computing.

[37] Anderson M.J. (2014). Permutational Multivariate Analysis of Variance (PERMANOVA). Wiley StatsRef: Statistics Reference Online.

[38] Liang Z., Zhang J., Ahmad A.A., Han J., Gharechahi J., Du M., Zheng J., Wang P., Yan P., Salekdeh G.H. (2023). Forage Lignocellulose Is an Important Factor in Driving the Seasonal Dynamics of Rumen Anaerobic Fungi in Grazing Yak and Cattle. Microbiol. Spectr..

[39] Gharechahi J., Vahidi M.F., Bahram M., Han J.-L., Ding X.-Z., Salekdeh G.H. (2021). Metagenomic Analysis Reveals a Dynamic Microbiome with Diversified Adaptive Functions to Utilize High Lignocellulosic Forages in the Cattle Rumen. ISME J..

[40] Aricha H., Simujide H., Wang C., Zhang J., Lv W., Jimisi X., Liu B., Chen H., Zhang C., He L. (2021). Comparative Analysis of Fecal Microbiota of Grazing Mongolian Cattle from Different Regions in Inner Mongolia, China. Animals.

[41] Sheikh A., Almathen F., Alfattah M. (2022). The Impact of Dromedary Camel Milk on Mice Gut Microbiota. Appl. Biol. Chem..

[42] Mahayri T.M., Fliegerová K.O., Mattiello S., Celozzi S., Mrázek J., Mekadim C., Sechovcová H., Kvasnová S., Atallah E., Moniello G. (2022). Host Species Affects Bacterial Evenness, but Not Diversity: Comparison of Fecal Bacteria of Cows and Goats Offered the Same Diet. Animals.

[43] Cunha I.S., Barreto C.C., Costa O.Y.A., Bomfim M.A., Castro A.P., Kruger R.H., Quirino B.F. (2011). Bacteria and Archaea Community Structure in the Rumen Microbiome of Goats (*Capra hircus*) from the Semiarid Region of Brazil. Anaerobe.

[44] St-Pierre B., Wright A.-D.G. (2012). Molecular Analysis of Methanogenic Archaea in the Forestomach of the Alpaca (*Vicugna pacos*). BMC Microbiol..

[45] Kumar S., Indugu N., Vecchiarelli B., Pitta D.W. (2015). Associative Patterns among Anaerobic Fungi, Methanogenic Archaea, and Bacterial Communities in Response to Changes in Diet and Age in the Rumen of Dairy Cows. Front. Microbiol..

[46] Fouts D.E., Szpakowski S., Purushe J., Torralba M., Waterman R.C., MacNeil M.D., Alexander L.J., Nelson K.E. (2012). Next Generation Sequencing to Define Prokaryotic and Fungal Diversity in the Bovine Rumen. PLoS ONE.

[47] Faridi F., Sena D.S., Sharma V. (2017). Characterisation of the Methanogenic Archaeal Community in the C1 Compartment of the Camel (*Camelus dromedarius*). J. Camel Pract. Res..

[48] Barelli C., Albanese D., Stumpf R.M., Asangba A., Donati C., Rovero F., Hauffe H.C. (2020). The Gut Microbiota Communities of Wild Arboreal and Ground-Feeding Tropical Primates Are Affected Differently by Habitat Disturbance. mSystems.

[49] Lv Q.-B., Meng J.-X., Ma H., Liu R., Qin Y., Qin Y.-F., Geng H.-L., Ni H.-B., Zhang X.-X. (2023). Description of Gut Mycobiota Composition and Diversity of Caprinae Animals. Microbiol. Spectr..

[50] Song J., Jeong J.Y., Kim M. (2017). Diversity Census of Fungi in the Ruminal Microbiome: A Meta-Analysis. J. Korea Acad. Ind. Coop. Soc..

[51] Comtet-Marre S., Parisot N., Lepercq P., Chaucheyras-Durand F., Mosoni P., Peyretaillade E., Bayat A.R., Shingfield K.J., Peyret P., Forano E. (2017). Metatranscriptomics Reveals the Active Bacterial and Eukaryotic Fibrolytic Communities in the Rumen of Dairy Cow Fed a Mixed Diet. Front. Microbiol..

[52] Accetto T., Avguštin G. (2019). The Diverse and Extensive Plant Polysaccharide Degradative Apparatuses of the Rumen and Hindgut Prevotella Species: A Factor in Their Ubiquity?. Syst. Appl. Microbiol..

[53] Portincasa P., Bonfrate L., Vacca M., De Angelis M., Farella I., Lanza E., Khalil M., Wang D.Q.-H., Sperandio M., Di Ciaula A. (2022). Gut Microbiota and Short Chain Fatty Acids: Implications in Glucose Homeostasis. Int. J. Mol. Sci..

[54] Daugherty M.S., Galyean M.L., Hallford D.M., Hageman J.H. (1986). Vitamin B12 and Monensin Effects on Performance, Liver and Serum Vitamin B12 Concentrations and Activity of Propionate Metabolizing Hepatic Enzymes in Feedlot Lambs. J. Anim. Sci..

[55] Strobel H.J. (1992). Vitamin B12-Dependent Propionate Production by the Ruminal Bacterium Prevotella Ruminicola 23. Appl. Environ. Microbiol..

[56] Purushe J., Fouts D.E., Morrison M., White B.A., Mackie R.I., Coutinho P.M., Henrissat B., Nelson K.E., North American Consortium for Rumen Bacteria (2010). Comparative Genome Analysis of *Prevotella ruminicola* and *Prevotella bryantii*: Insights into Their Environmental Niche. Microb. Ecol..

[57] de Oliveira Melo N.C., Cuevas-Sierra A., Fernández-Cruz E., de la O V., Martínez J.A. (2023). Fecal Microbiota Composition as a Metagenomic Biomarker of Dietary Intake. Int. J. Mol. Sci..

[58] Mukherjee A., Lordan C., Ross R.P., Cotter P.D. (2020). Gut Microbes from the Phylogenetically Diverse Genus Eubacterium and Their Various Contributions to Gut Health. Gut Microbes.

[59] Kasai C., Sugimoto K., Moritani I., Tanaka J., Oya Y., Inoue H., Tameda M., Shiraki K., Ito M., Takei Y. (2016). Comparison of Human Gut Microbiota in Control Subjects and Patients with Colorectal Carcinoma in Adenoma: Terminal Restriction Fragment Length Polymorphism and next-Generation Sequencing Analyses. Oncol. Rep..

[60] Janssen P.H., Kirs M. (2008). Structure of the Archaeal Community of the Rumen. Appl. Environ. Microbiol..

[61] Chaucheyras-Durand F., Masséglia S., Fonty G., Forano E. (2010). Influence of the Composition of the Cellulolytic Flora on the Development of Hydrogenotrophic Microorganisms, Hydrogen Utilization, and Methane Production in the Rumens of Gnotobiotically Reared Lambs. Appl. Environ. Microbiol..

[62] Abecia L., Martín-García A.I., Martínez G., Newbold C.J., Yáñez-Ruiz D.R. (2013). Nutritional Intervention in Early Life to Manipulate Rumen Microbial Colonization and Methane Output by Kid Goats Postweaning. J. Anim. Sci..

[63] Rabee A.E., Forster R.J., Elekwachi C.O., Kewan K.Z., Sabra E.A., Shawket S.M., Mahrous H.A., Khamiss O.A. (2019). Community Structure and Fibrolytic Activities of Anaerobic Rumen Fungi in Dromedary Camels. J. Basic Microbiol..

[64] Sridhar M., Kumar D., Anandan S., Prasad C., Sampath K. (2007). Occurrence and Prevalence of Cyllamyces Genus—A Putative Anaerobic Gut Fungus in Indian Cattle and Buffaloes. Curr. Sci..

[65] Ozkose E., Thomas B.J., Davies D.R., Griffith G.W., Theodorou M.K. (2001). *Cyllamyces aberensis* Gen.Nov. Sp.Nov., a New Anaerobic Gut Fungus with Branched Sporangiophores Isolated from Cattle. Can. J. Bot..

[66] Sadek A., Taminiau B., Daube G., Sapountzis P., Chaucheyras-Durand F., Castex M., Coucheney F., Drider D. (2024). Impact of Dietary Regime and Seasonality on Hindgut’s Mycobiota Diversity in Dairy Cows. Microorganisms.

[67] Yi S., Wu H., Liu Y., Dai D., Meng Q., Chai S., Liu S., Zhou Z. (2023). Concentrate Supplementation Improves Cold-Season Environmental Fitness of Grazing Yaks: Responsive Changes in the Rumen Microbiota and Metabolome. Front. Microbiol..

[68] Treon E., Sidney T., Taiwo G., Idowu M., Leal Y., Ologunagba D., Ogunade I.M. (2024). Effects of Dietary Supplementation of a Blend of Saccharomyces Cerevisiae, Multiple Live Probiotic Bacteria, and Their Fermentation Products on Performance, Health, and Rumen Bacterial Community of Newly Weaned Beef Steers during a 56-d Receiving Period. Transl. Anim. Sci..

[69] Spatz M., Richard M.L. (2020). Overview of the Potential Role of Malassezia in Gut Health and Disease. Front. Cell. Infect. Microbiol..

[70] Ianiri G., LeibundGut-Landmann S., Dawson T.L. (2022). Malassezia: A Commensal, Pathogen, and Mutualist of Human and Animal Skin. Annu. Rev. Microbiol..

[71] Kišidayová S., Durkaj D., Mihaliková K., Váradyová Z., Puchalska J., Szumacher-Strabel M., Cieślak A., Gizejewski Z. (2021). Rumen Ciliated Protozoa of the Free-Living European Bison (*Bison bonasus*, Linnaeus). Front. Microbiol..

[72] Huuki H., Tapio M., Mäntysaari P., Negussie E., Ahvenjärvi S., Vilkki J., Vanhatalo A., Tapio I. (2022). Long-Term Effects of Early-Life Rumen Microbiota Modulation on Dairy Cow Production Performance and Methane Emissions. Front. Microbiol..

[73] Fregulia P., Campos M.M., Dias R.J.P., Liu J., Guo W., Pereira L.G.R., Machado M.A., de Lima Reis Faza D.R., Guan L.L., Garnsworthy P.C. (2022). Taxonomic and Predicted Functional Signatures Reveal Linkages between the Rumen Microbiota and Feed Efficiency in Dairy Cattle Raised in Tropical Areas. Front. Microbiol..

[74] Villafuerte A., Haro A.N., Andrade M.J., Fiallos L., Bautista H. (2023). Response of Hematological Parameters in Breeding Male Alpacas from High Andean Zones. J. Surv. Fish. Sci..

[75] Zhao P., Li S., He Z., Ma X. (2024). Physiological and Genetic Basis of High-Altitude Indigenous Animals’ Adaptation to Hypoxic Environments. Animals.

[76] Quagliariello A., Di Paola M., De Fanti S., Gnecchi-Ruscone G.A., Martinez-Priego L., Perez-Villaroya D., Sherpa M.G., Sherpa P.T., Marinelli G., Natali L. (2019). Gut Microbiota Composition in Himalayan and Andean Populations and Its Relationship with Diet, Lifestyle and Adaptation to the High-Altitude Environment. J. Anthropol. Sci..

[77] Correia Sales G.F., Carvalho B.F., Schwan R.F., de Figueiredo Vilela L., Moreno Meneses J.A., Gionbelli M.P., da Silva Ávila L.C. (2021). Heat Stress Influence the Microbiota and Organic Acids Concentration in Beef Cattle Rumen. J. Therm. Biol..

[78] Maynez-Perez A., Jahuey-Martínez F.J., Martínez-Quintana J.A., Hume M.E., Anderson R.C., Corral-Luna A., Rodríguez-Almeida F.A., Castillo-Castillo Y., Felix-Portillo M. (2024). The Rumen Microbiome Composition of Raramuri Criollo and European Cattle in an Extensive System. Microorganisms.

[79] Chao R., Xia C., Pei C., Huo W., Liu Q., Zhang C., Ren Y. (2021). Comparison of the Microbial Communities of Alpacas and Sheep Fed Diets with Three Different Ratios of Corn Stalk to Concentrate. J. Anim. Physiol. Anim. Nutr..

[80] Chellapandi P., Bharathi M., Sangavai C., Prathiviraj R. (2018). Methanobacterium Formicicum as a Target Rumen Methanogen for the Development of New Methane Mitigation Interventions: A Review. Vet. Anim. Sci..

