# Supplementary material for: Effects of Diet and Altitude on the Microbiota of the First Compartment of the Stomach in Peruvian Alpacas in Highland Puna Regions and Their Correlations with Blood Parameters
Source: Microorganisms. 2026 Jan 8;14(1):138. doi: 10.3390/microorganisms14010138 (PMC12844463; doi:10.3390/microorganisms14010138)
Supplement: Supplementary file 1 [file microorganisms-14-00138-s001.zip › Supplementary Figure.pdf]

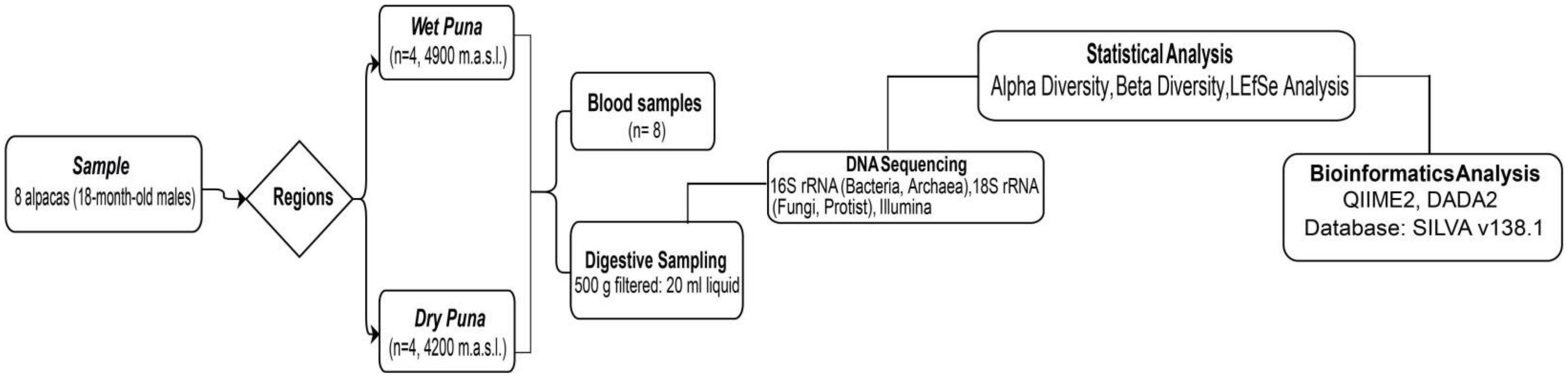


Figure S1. Flowchart of the procedures used in this study.

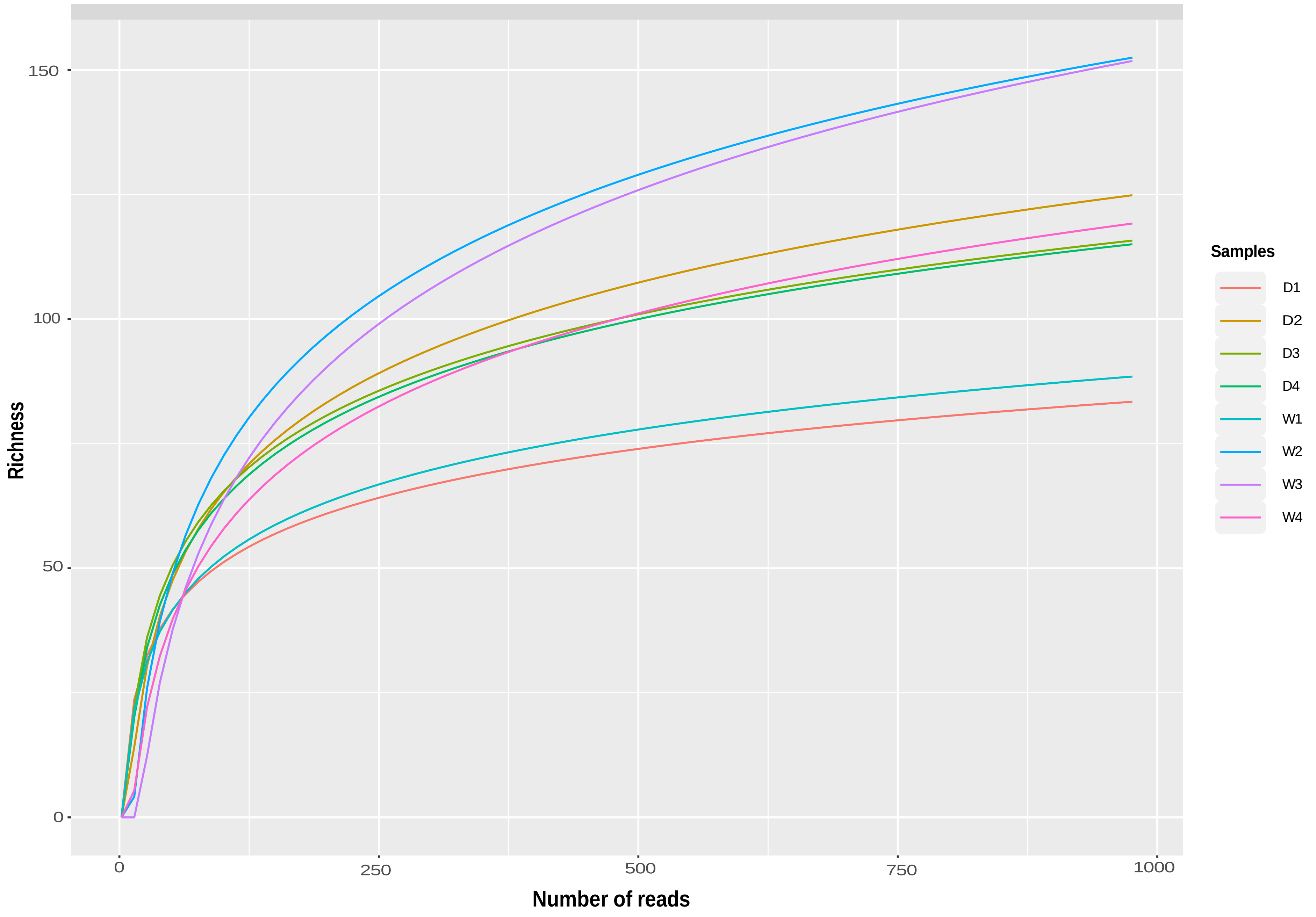


Figure S2: Species richness rarefaction curves show sequencing depth of 16S data obtained from bacteria from compartiment 1 samples.

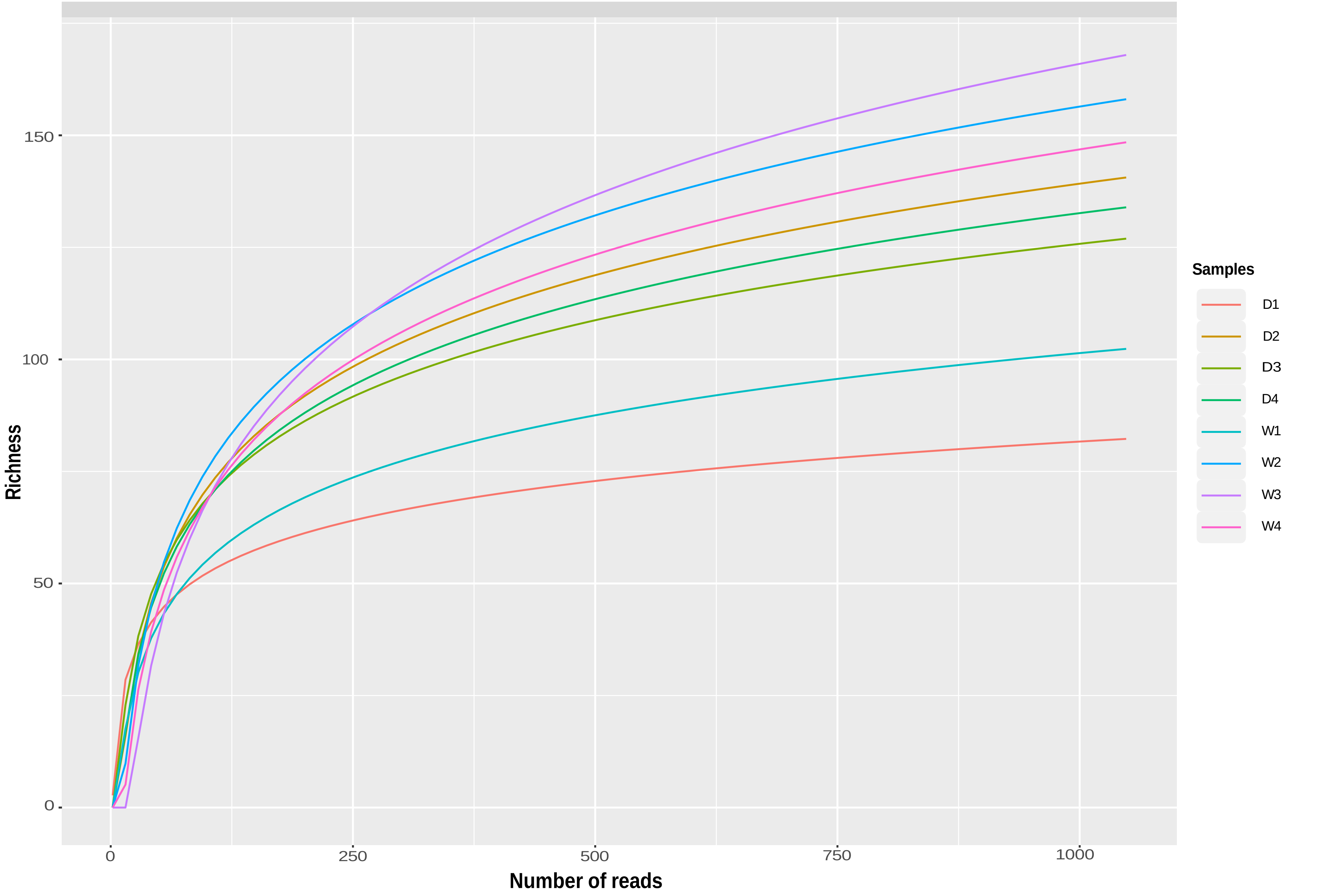


Figure S3: Species richness rarefaction curves show sequencing depth of 16S data obtained from archae from compartiment 1 samples.

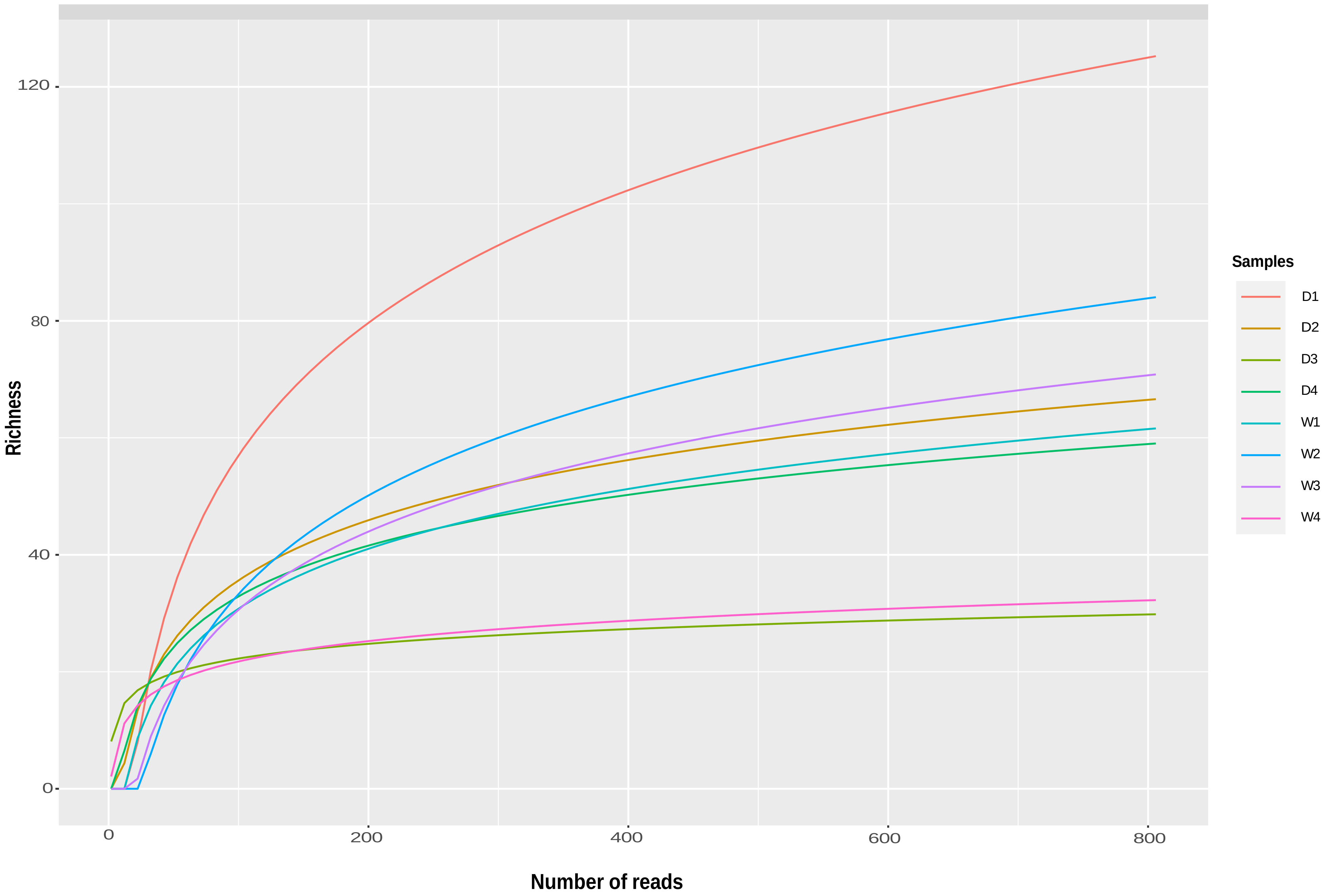


Figure S4: Species richness rarefaction curves show sequencing depth of 18S data obtained from fungi from compartiment 1 samples.

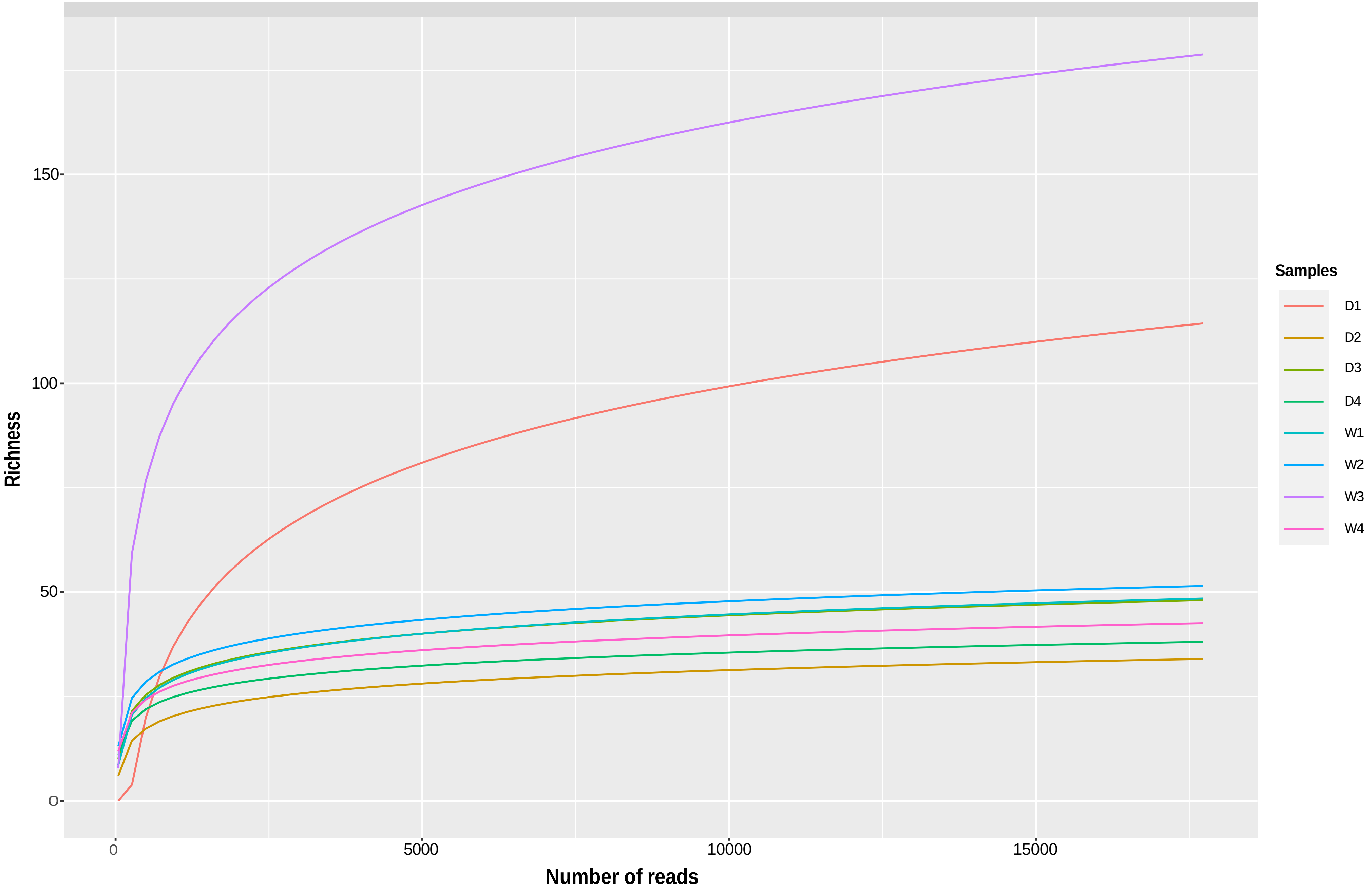


Figure S5: Species richness rarefaction curves show sequencing depth of 18S data obtained from protist from compartiment 1 samples.

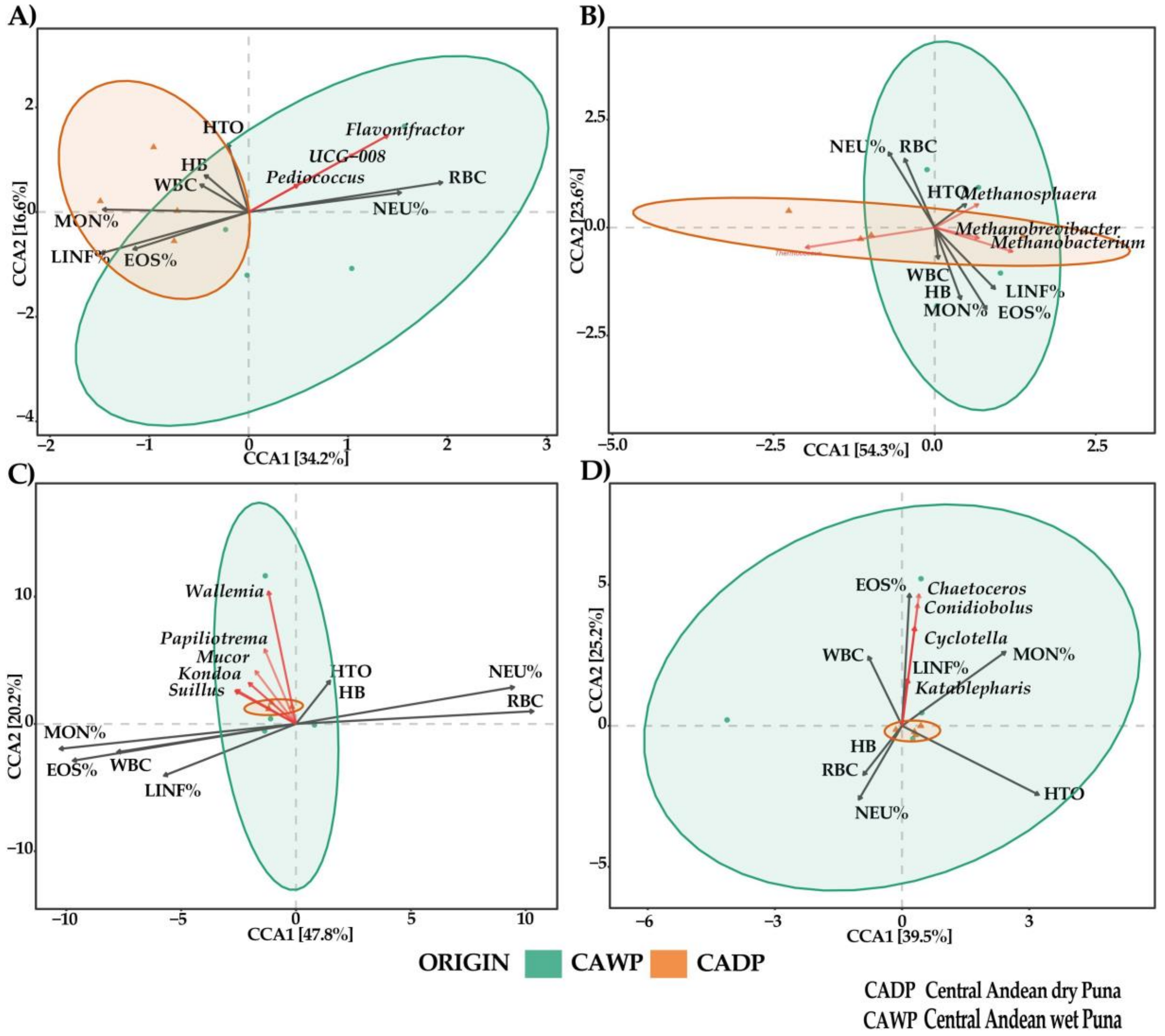


Figure S6: Canonical correlation analysis for Origin. A) Bacteria CCA. B) Archaea CCA. C) Fungi CCA. D) Protist CCA.
